# Prognostic value and immune infiltration of novel signatures in clear cell renal cell carcinoma microenvironment

**DOI:** 10.18632/aging.102233

**Published:** 2019-09-07

**Authors:** Wen-Hao Xu, Yue Xu, Jun Wang, Fang-Ning Wan, Hong-Kai Wang, Da-Long Cao, Guo-Hai Shi, Yuan-Yuan Qu, Hai-Liang Zhang, Ding-Wei Ye

**Affiliations:** 1 Department of Urology, Fudan University Shanghai Cancer Center, Shanghai 200032, P.R. China; 2 Department of Oncology, Shanghai Medical College, Fudan University, Shanghai 200032, P.R. China; 3 Department of Ophthalmology, The First Affiliated Hospital of Soochow University, Suzhou 215000, P.R. China

**Keywords:** clear cell renal cell carcinoma, tumor microenvironment, ESTIMATE algorithm, immune signature, prognosis

## Abstract

Growing evidence has highlighted the immune response as an important feature of carcinogenesis and therapeutic efficacy in clear cell renal cell carcinoma (ccRCC). This study categorized ccRCC cases into high and low score groups based on their immune/stromal scores generated by the ESTIMATE algorithm, and identified an association between these scores and prognosis. Differentially expressed tumor environment (TME)-related genes extracted from common upregulated components in immune and stromal scores were described using functional annotations and protein–protein interaction (PPI) networks. Most PPIs were selected for further prognostic investigation. Many additional previously neglected signatures, including *AGPAT9, AQP7, HMGCS2, KLF15, MLXIPL, PPARGC1A*, exhibited significant prognostic potential. In addition, multivariate Cox analysis indicated that *MIXIPL* and *PPARGC1A* were the most significant prognostic signatures, and were closely related to immune infiltration in TCGA cohort. External prognostic validation of *MIXIPL* and *PPARGC1A* was undertaken in 380 ccRCC cases from a real-world cohort. These findings indicate the relevance of monitoring and manipulation of the microenvironment for ccRCC prognosis and precision immunotherapy.

## INTRODUCTION

Renal cell carcinoma (RCC) has become one of the most common genitourinary tumors, with an estimated 73,820 new cases and 14,770 deaths occurring in the United States in 2019 [1]. RCC incidence accounts for approximately 5% of new cancer cases in males and 3% of female cases [1]. As the major subtype of kidney cancer, clear cell renal cell carcinoma (ccRCC) is one of the most malignant urinary tumors with a global annual mortality rate of approximately 90,000 [2]. Metastasis is found in 25%–30% patients at initial diagnosis of ccRCC. Cytokine and checkpoint inhibitor immunotherapy have been demonstrated to promote active immune responses via different mechanisms, including genetic aberrations, epithelial–mesenchymal transition, and metabolism [3, 4]. Although extensive researches have been conducted on the mechanisms of cancer development and progression, the etiology and carcinogenesis of ccRCC remain unclear [5]. Therefore, considering the high morbidity and mortality of ccRCC, it is essential to explore molecular signatures with prognostic value that affect immune response in ccRCC patients.

The tumor microenvironment (TME) is a mixture of fluids, immune cells, stromal cells, extracellular matrix molecules, and numerous cytokines and chemokines. The cells and molecules in the TME are in a dynamic process, reflecting the evolutionary nature of cancer, and jointly promote tumor immune escape, tumor growth and metastasis [6]. Although multiple genetic mutations increase the incidence of cancer, researchers are not aware of the impact of the TME on tumor progression or immune response [7]. Li reviewed that the TME can impose metabolic stress on immune cell infiltration, leading to local immunosuppression and limited reinvigoration of antitumor immunity [8]. Therefore, understanding the molecular composition and function of the TME is critical to effectively manage cancer progression and immune response [9–11].

Bioinformatics analysis generates large and complex biological data through the comprehensive use of biology, computer science, and information technology. Its rapid development, such as The Cancer Genome Atlas (TCGA) database, provides researchers with a more user-friendly and convenient platform, guiding the implementation of basic experiments [12, 13]. In 2013, Yoshihara et al. calculated specific molecular biomarker expression in immune and stromal cells, and thus generated an ESTIMATE algorithm with immune/stromal/ESTIMATE scores to predict the TME [9]. Based on the ESTIMATE algorithm, researchers have performed prognostic assessments and exploration of genetic alterations in many neoplasms [11, 14, 15]. However, the value of immune/stromal scores for ccRCC remains to be elucidated.

In this current work, to investigate potential signatures for ccRCC patients, we obtained a list of TME-related genes of prognostic value using immune/stromal scores after ESTIMATE algorithm-processing in multiple cohorts. Functional annotations and immune infiltration correlation were analyzed for significant hub genes. We hypothesized that the possible oncogenic activity of hub genes correlates with poor prognosis and might reveal potential immune therapies by providing insights into the molecular mechanisms of ccRCC.

## RESULTS

### Elevated immune and stromal score significantly correlated with advanced clinicopathological indicators and poor prognosis

**Figure 1:**
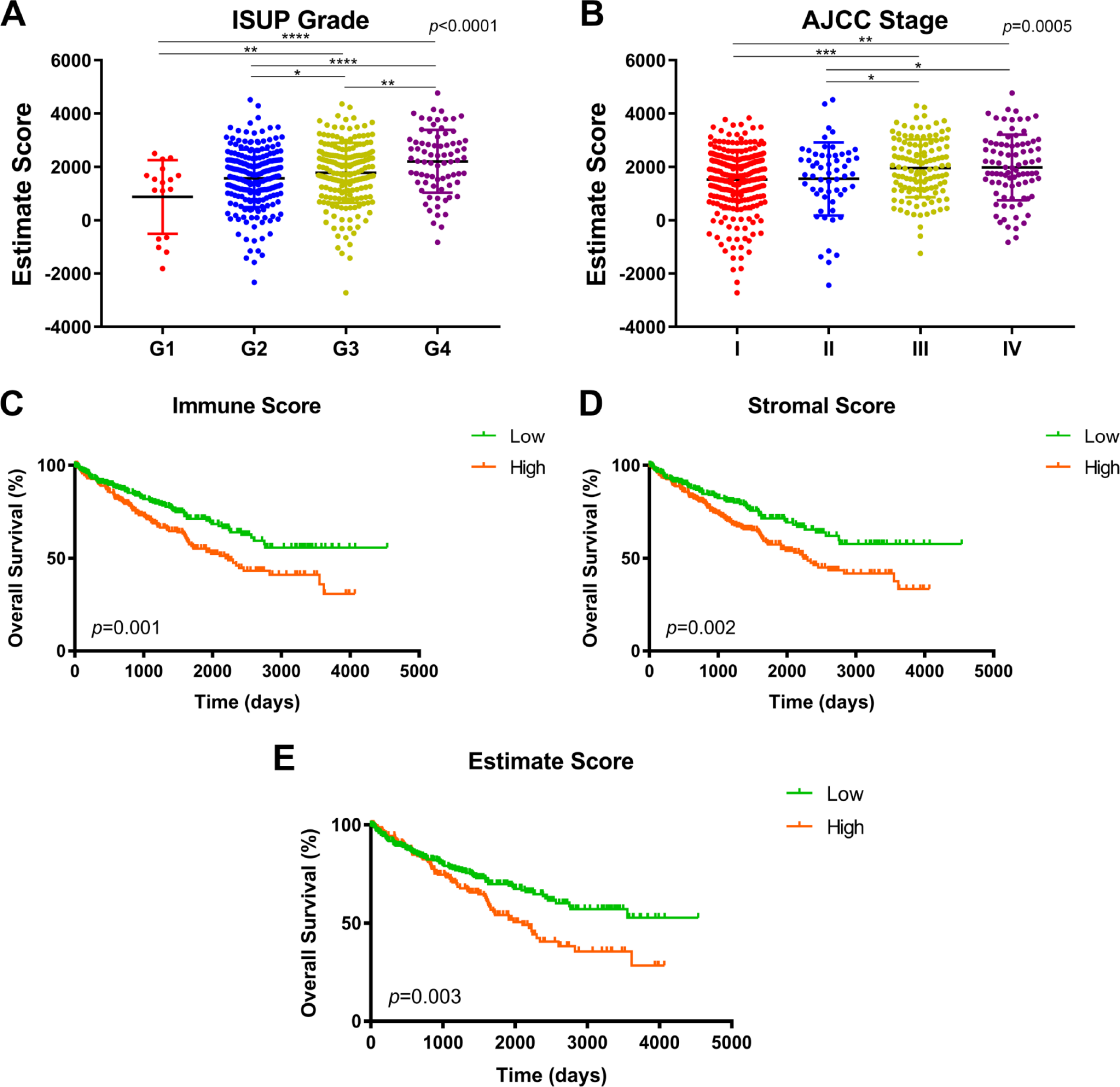
**Association between immune/stromal/Estimate score and prognosis in TCGA after ESTIMATE algorithm processed.** (**A**–**B**) Estimate score was significantly associated with higher ISUP grade and AJCC stage (p<0.001). The highest Estimate score was found in the most progressive clinicopathological stage, G4 and stage IV. (**C**) Survival curves indicated that elevated immune score significantly correlated with poor overall survival in 533 ccRCC patients (p=0.001, 1165 vs. 1217 days). (**D**) Increased stromal score significantly associated with shorter OS (p=0.002, 1117.5 vs. 1230 days). (**E**) Significant Estimate score also predict significant OS for ccRCC patients (p=0.003, 1172.5 vs. 1223.5 days).

Transcriptional expression profiles and phenotype data was download and integrated in 533 ccRCC patients from TCGA cohort. 64.7% (n=345) patients were male and 35.5% (n=188) were female. T1-T2 stage patients accounted for 64.1% (n=342) of the total number. N0 and N1 patients accounted for 45% (n=240) and 3% (n=16), respectively. M0 and M1 patients accounted for 79.2% (n=422) and 14.8% (n=79). In addition, after ESTIMATE algorithm was processed, stromal scores and immune scores were obtained, ranging from -2,716.84 to 4,773.7 and -1,158.94 to 3,076.7, respectively. Estimate score was significantly associated with higher ISUP grade and AJCC stage (Figure 1A, p<0.001, Figure 1B, p=0.0005). The highest Estimate score was found in the most progressive clinicopathological stage, G4 and stage IV. Immune score indicated significant prognostic implications, associated with elevated ISUP grade and AJCC stage (Supplementary Figure 1A–1B, p<0.0001). Stromal score significantly correlated with advanced ISUP grade (Supplementary Figure 1C, p=0.0463), while showed no association with AJCC stage (Supplementary Figure 1D, p=0.0674).

To detect potential correlation between immune/stromal/Estimate score and survival benefits, we divided 533 patients into high and low score groups. Survival curves indicated that elevated immune score significantly correlated with poor overall survival (Figure 1C; *p*=0.001, 1165 vs. 1217 days). Increased stromal score significantly associated with shorter OS (Figure 1D; *p*=0.002, 1117.5 vs. 1230 days). Significant Estimate score also predict significant OS for ccRCC patients (Figure 1E; *p*=0.003, 1172.5 vs. 1223.5 days).

### Differential expressed genes with immune and stromal score in ccRCC

**Figure 2:**
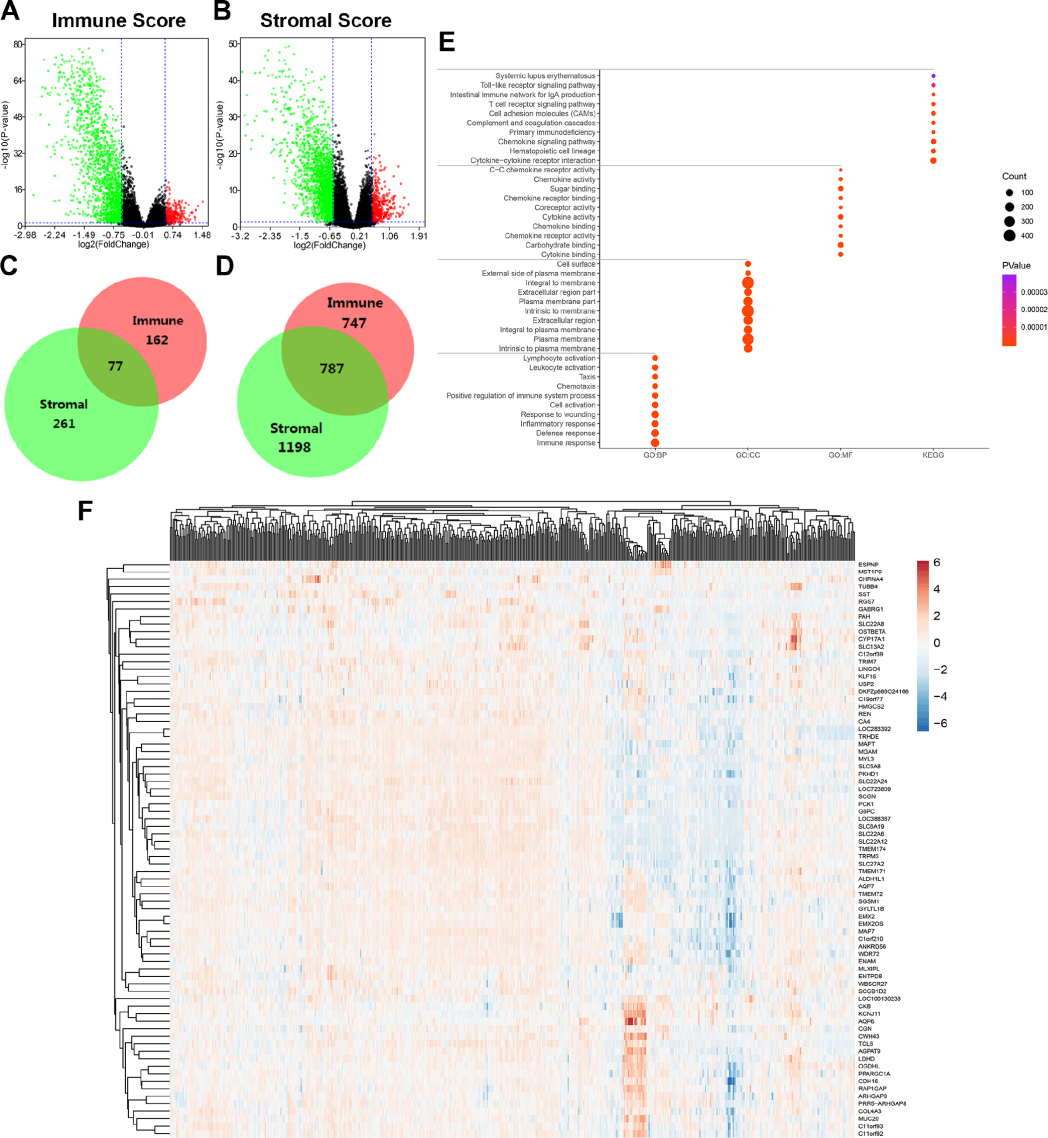
**Differential expressed genes with immune and stromal score and related functional annotations in ccRCC.** (**A**) Based on immune score comparison, 162 genes were up-regulated and 747 genes down-regulated in the high score than the low score group after propensity analysis using limma package algorithm. (**B**) Similarly, for high stromal score compared with low score, 261 up-regulated genes and 1198 down-regulated genes were obtained. (**C**–**D**) A total of 77 DEGs were commonly upregulated in the high scores groups, and 787 genes were synchronously downregulated using Venn algorithm. (**E**) functional enrichment analysis including GO: BP, GO: CC, GO: MF and KEGG pathways, was performed in 864 commonly DEGs. (**F**) Cluster analysis and heat map including 77 up-regulated DEGs suggested distinct mRNA expression profiles of DEGs in 533 ccRCC samples.

To explore differential expressed genes (DEGs) profiles with immune and/or stromal scores, we performed transcriptional microarray analysis of 533 ccRCC cases from TCGA cohort. Based on immune score comparison, 162 genes were up-regulated and 747 genes down-regulated in the high score than the low score group after propensity analysis using limma package algorithm (Figure 2A). Similarly, for high stromal score compared with low score, 261 up-regulated genes and 1198 down-regulated genes were obtained (Figure 2B). A total of 77 DEGs were commonly upregulated in the high scores groups (Figure 2C), and 787 genes were synchronously downregulated using Venn algorithm (Figure 2D).

In addition, functional enrichment analysis including GO: BP, GO: CC, GO: MF and KEGG pathways, was performed in 864 commonly DEGs in Figure 2E. After –Log (FDR) sorting, we listed the top 10 function annotations of each part. As illustrated in Supplementary Figure 2, DEGs were mostly enriched in immune defense, plasma membrane, cytokine binding and cytokine-cytokine receptor interaction. Cluster analysis and heat map including 77 up-regulated DEGs suggested distinct mRNA expression profiles of DEGs in 533 ccRCC samples (Figure 2F).

### Significant modular analysis based on PPI network

**Figure 3:**
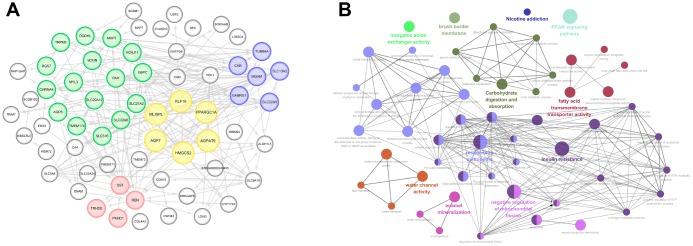
**Significant modular analysis and function enrichment analysis based on PPI network.** (**A**) PPI network was constructed using a total of 77 commonly up-regulated DEGs. MCODE, plug-in of Cytoscape, was used to detect most significant co-regulated modular. The most significant modular including AGPAT9, AQP7, HMGCS2, KLF15, MLXIPL and PPARGC1A, was marked in yellow. (**B**) functional annotations indicated that 77 DEGs were mostly involved in carbohydrate digestion and absorption, fatty acid transmembrane transport activity, PPAP signaling pathway, response to methionine, insulin resistance, water channel activity, enamel mineralization, negative regulation of mitochondrial fission, etc.

PPI network was constructed using a total of 77 commonly up-regulated DEGs in Figure 3A. MCODE, plug-in of Cytoscape, was used to detect most significant co-regulated modular. It indicated four closely related subgroups displayed in different color, and the most significant modular including *AGPAT9, AQP7, HMGCS2, KLF15, MLXIPL, PPARGC1A*, was marked in yellow. Survival curves of other nodes were illustrated in Supplementary Figure 3. It suggested that decreased *SLC27A2, G6PC, MGAM, TRPM3, PKHD1, MYL3, MAPT, SLC22A6, TRHDE, TMEM174, SLC22A8, OGDHL, SCGN, SLC51B, SLC22A12, REN, PAH, GABRG1, SLC13A2, SST, KCNJ11* significantly correlated with poor OS, while elevated *TUBB4A* and *RGS7* expression significantly predicted poor prognosis (*p*<0.05).

As shown in Figure 3B, functional annotations indicated that 77 DEGs were mostly involved in carbohydrate digestion and absorption, fatty acid transmembrane transport activity, PPAP signaling pathway, response to methionine, insulin resistance, water channel activity, enamel mineralization, negative regulation of mitochondrial fission, etc.

### Survival analysis of significant DEGs in ccRCC from TCGA database

After integrating mRNA expression profile of six significant hub genes (*AGPAT9, AQP7, HMGCS2, KLF15, MLXIPL, PPARGC1A*) and clinical information, univariate regression analysis of overall survival were performed in TCGA cohort. As shown in Table 1, stromal score (ref. low), Immune score (ref. low), pT stage (ref. T1-T2), pN stage (ref. N0), pM stage (ref. M0), AJCC stage (ref. I-II), ISUP grade (ref. 1-2), *AGPAT9, AQP7, HMGCS2, KLF15, MLXIPL* and *PPARGC1A* expression (ref. low) were demonstrated as independent prognostic indicators for ccRCC patients (p<0.05). Multivariate Cox analysis showed that poor OS was significantly associated with pM stage (ref. M0; HR=2.807, *p*<0.001), ISUP grade (ref. 1-2; HR=1.765, *p*=0.029), *MLXIPL* expression (ref. low; HR=2.537, *p*=0.005) and *PPARGC1A* expression (ref. low; HR=0.468, *p*=0.009).

**Table 1:** Univariate and multivariate Cox logistic regression analysis of overall survival in TCGA cohort.

**Covariates**	**Univariate analysis**				**Multivariate analysis**		
	**HR**	**95% CI**	***P* value**		**HR**	**95% CI**	***P* value**
Stromal score (ref. low)	1.459	1.070-1.990	**0.017**		1.512	0.908-2.516	0.112
Immune score (ref. low)	1.628	1.207-2.196	**0.001**		1.092	0.688-1.731	0.710
pT stage (ref. T1-T2)	3.155	2.332-4.268	**<0.001**		1.274	0.549-2.954	0.573
pN stage (ref. N0)	2.887	1.535-5.429	**0.001**		1.265	0.636-2.516	0.503
pM stage (ref. M0)	4.396	3.234-5.974	**<0.001**		2.807	1.606-4.908	**<0.001**
AJCC stage (ref. I-II)	3.856	2.814-5.285	**<0.001**		1.276	0.495-3.287	0.614
ISUP grade (ref. 1-2)	3.056	2.166-4.311	**<0.001**		1.765	1.060-2.939	**0.029**
AGPAT9 expression (ref. low)	0.449	0.333-0.605	**<0.001**		0.832	0.500-1.384	0.479
AQP7 expression (ref. low)	0.551	0.407-0.746	**<0.001**		1.150	0.694-1.903	0.588
HMGCS2 expression (ref. low)	0.487	0.362-0.656	**<0.001**		0.883	0.550-1.418	0.607
KLF15 expression (ref. low)	0.567	0.409-0.787	**0.001**		0.782	0.461-1.328	0.363
MLXIPL expression (ref. low)	1.893	1.246-2.875	**0.003**		2.537	1.333-4.827	**0.005**
PPARGC1A expression (ref. low)	0.288	0.206-0.405	**<0.001**		0.468	0.264-0.828	**0.009**

TCGA: the Cancer Genome Atlas.

**Figure 4:**
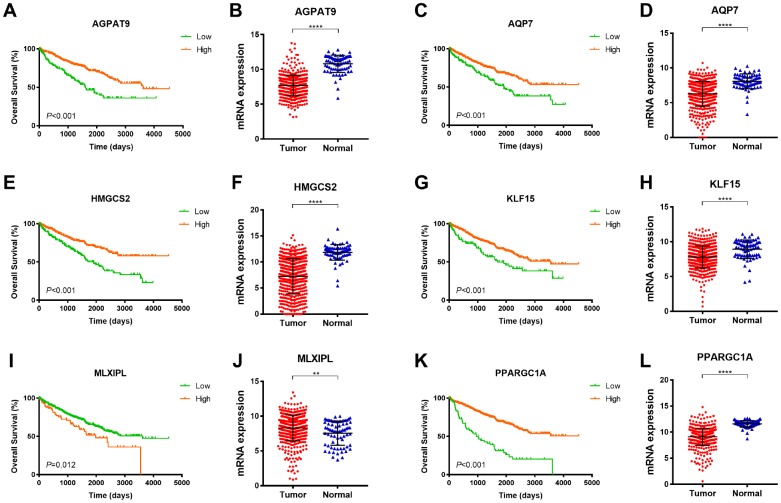
**Survival analysis of significant DEGs in 533 ccRCC from TCGA database.** Among 6 significant hub genes, significantly decreased AGPAT9, AQP7, HMGCS2, KLF15, PPARGC1A mRNA expressions were found in ccRCC tissues compared with adjacent normal tissues, while MLXIPL mRNA expression was significantly elevated in tumor samples compared with normal samples. Kaplan-Meier method indicated that decreased AGPAT9, AQP7, HMGCS2, KLF15, PPARGC1A mRNA expression significantly correlated with poor OS (p<0.001), and elevated MLXIPL mRNA expression was significantly associated with shorter OS for ccRCC patients (p=0.012).

As demonstrated in Figure 4, among 6 significant hub genes, significantly decreased *AGPAT9, AQP7, HMGCS2, KLF15, PPARGC1A* mRNA expressions were found in ccRCC tissues compared with adjacent normal tissues, while *MLXIPL* mRNA expression was significantly elevated in tumor samples compared with normal samples. Kaplan-Meier method indicated that decreased *AGPAT9, AQP7, HMGCS2, KLF15, PPARGC1A* mRNA expression significantly correlated with poor OS (*p*<0.001), and elevated *MLXIPL* mRNA expression was significantly associated with shorter OS for ccRCC patients (*p*=0.012).

### Prognostic validation of MLXIPL and PPARGC1A in FUSCC cohort

**Figure 5:**
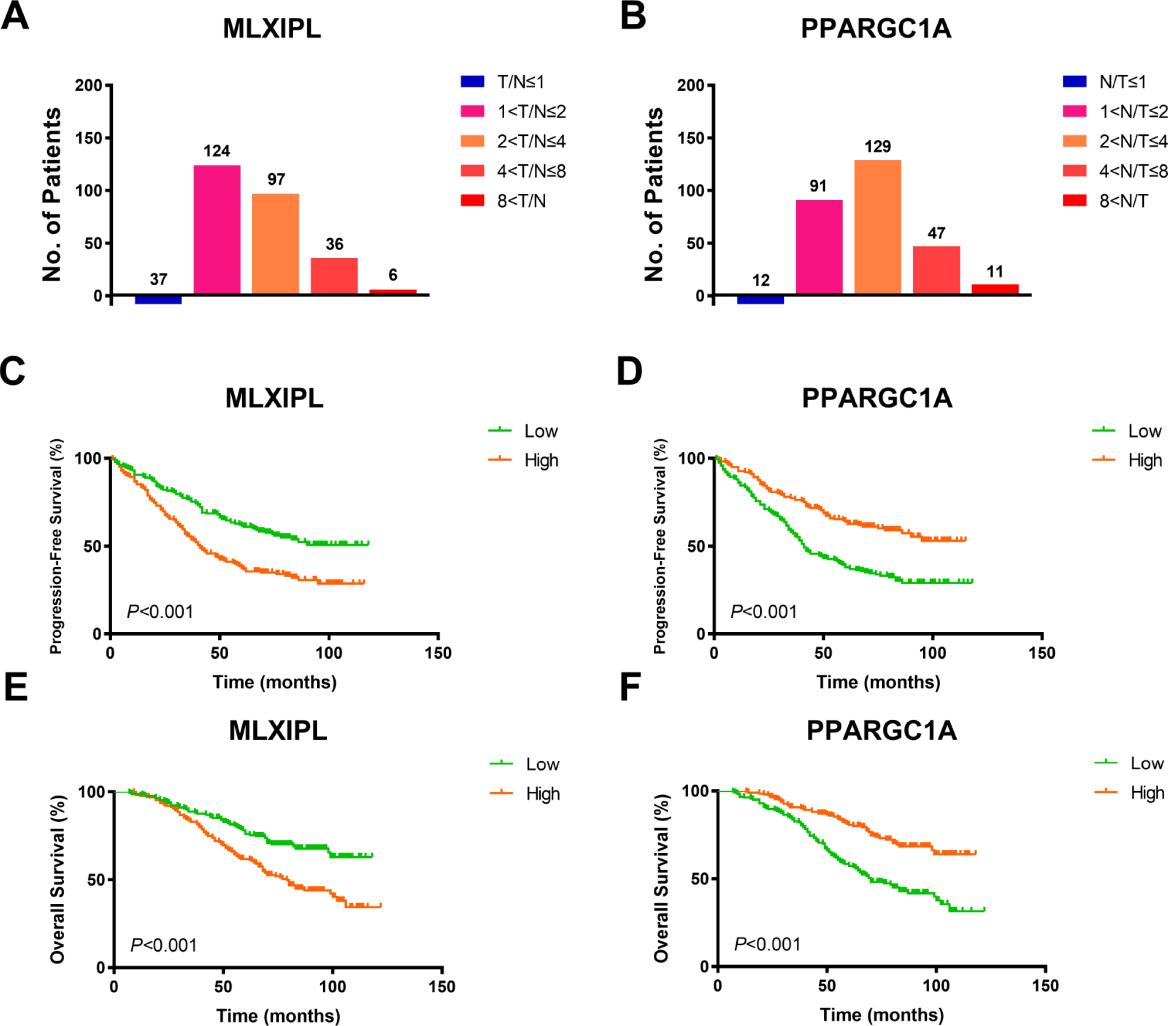
**Prognostic validation of MLXIPL and PPARGC1A in FUSCC cohort.** (**A**–**B**) To validate AQP9 mRNA expression profile in ccRCC tissues, we performed RT-qPCR using 380 paired tumor and normal samples with available clinical follow-up data from a real-world cohort. It revealed dramatically increased MLXIPL and decreased PPARGC1A mRNA expression in ccRCC samples than normal tissues. (**C**–**F**) Survival curves suggested that patients with elevated MLXIPL and decreased PPARGC1A mRNA levels significantly correlated with poorer PFS and OS (p<0.001).

To validate *AQP9* mRNA expression profile in ccRCC tissues, we performed RT-qPCR using 380 paired tumor and normal samples with available clinical follow-up data from a real-world cohort. It revealed dramatically increased *MLXIPL* and decreased *PPARGC1A* mRNA expression in ccRCC samples than normal tissues (Figure 5A–5B). Survival curves suggested that patients with elevated *MLXIPL* and decreased *PPARGC1A* mRNA levels significantly correlated with poorer PFS and OS (*p*<0.001; Figure 5C–5F).

### Cox regression analysis and ROC curves

Multivariate Cox regression analysis of PFS and OS were performed in FUSCC cohort using mRNA expression profile of *MLXIPL* and *PPARGC1A* and clinicopathological information. As shown in Table 2, multivariate Cox analysis showed that poor PFS and OS were significantly associated with pT stage (ref. T1-T2), pN stage (ref. N0), pM stage (ref. M0), AJCC stage (ref. I-II), ISUP grade (ref. 1-2) and gene (*MLXIPL* or *PPARGC1A*) expression (ref. low) for ccRCC patients of FUSCC cohort (*p*<0.05).

**Table 2:** Multivariate Cox logistic regression analysis of PFS and OS in FUSCC cohort.

**Covariates**	**MLXIPL**								**PPARGC1A**						
	**PFS**				**OS**				**PFS**				**OS**		
	**HR**	**95% CI**	***P* value**		**HR**	**95% CI**	***P* value**		**HR**	**95% CI**	***P* value**		**HR**	**95% CI**	***P* value**
pT stage (ref. T1-T2)	2.105	1.243-3.567	**0.006**		1.840	1.067-3.172	**0.028**		1.931	1.141-3.266	**0.014**		1.862	1.082-3.206	**0.025**
pN stage (ref. N0)	1.922	1.234-2.994	**0.004**		1.832	1.163-2.885	**0.009**		2.029	1.304-3.156	**0.002**		1.821	1.162-2.854	**0.009**
pM stage (ref. M0)	1.771	1.107-2.834	**0.017**		2.001	1.206-3.321	**0.007**		1.641	1.034-2.606	**0.036**		1.912	1.160-3.150	**0.011**
AJCC stage (ref. I-II)	2.413	1.244-4.679	**0.009**		3.434	1.723-6.845	**<0.001**		2.721	1.425-5.197	**0.002**		3.511	1.776-6.942	**<0.001**
ISUP grade (ref. 1-2)	1.934	1.419-2.636	**<0.001**		1.764	1.210-2.571	**0.003**		1.823	1.340-2.481	**<0.001**		1.749	1.199-2.551	**0.004**
Gene expression (ref. Low)	1.963	1.460-2.640	**<0.001**		1.545	1.102-2.166	**0.012**		0.524	0.379-0.726	**<0.001**		0.665	0.457-0.967	**0.033**

PFS: progression-free survival; OS: overall survival; FUSCC: Fudan University Shanghai Cancer Center

**Figure 6:**
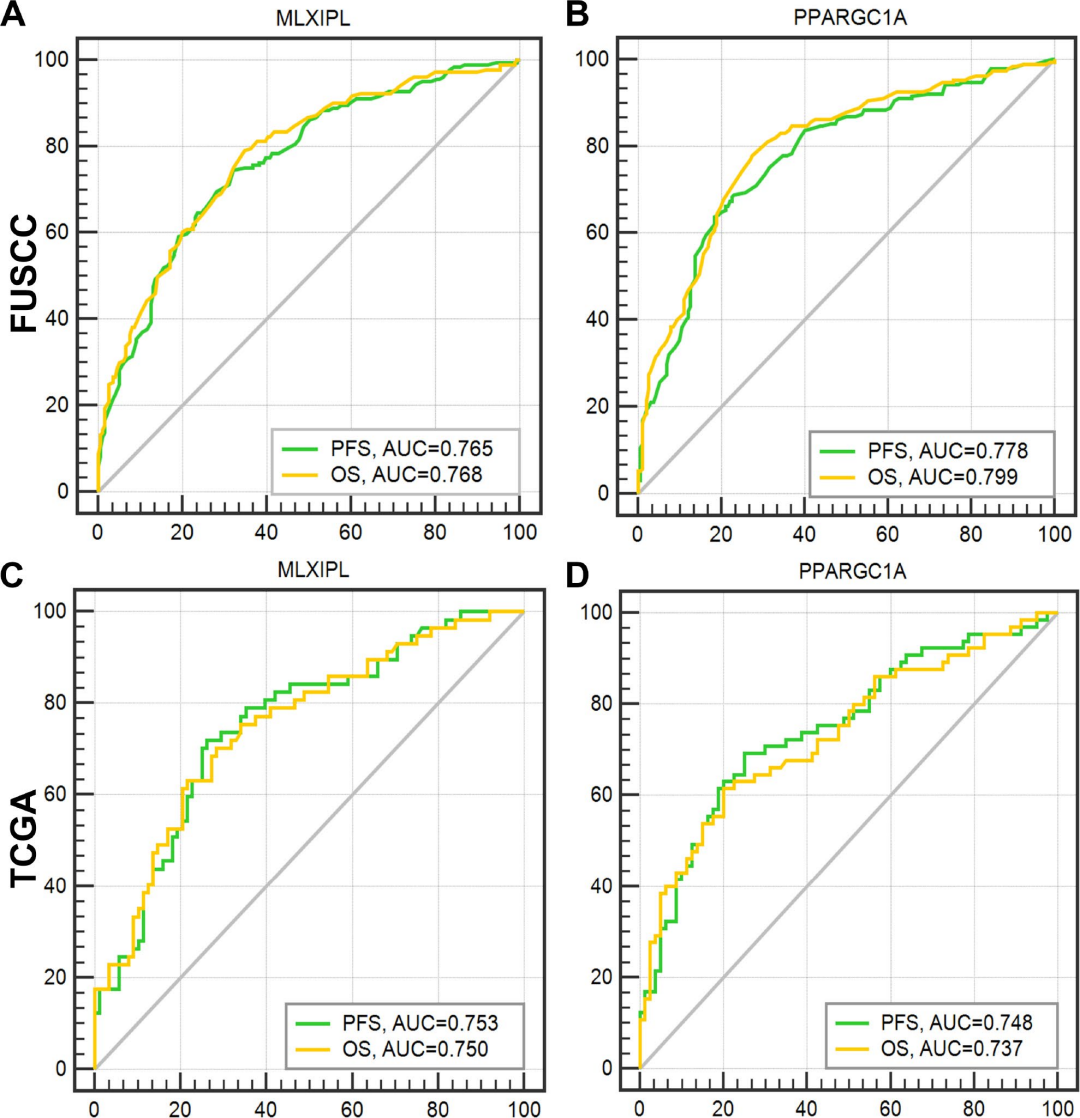
**ROC curves were generated to validate the ability of the logistic model to predict prognosis.** After integrating all the significant clinicopathological parameters and gene expression profiles in the multivariate Cox regression models of FUSCC cohort, we generated the formulas for MLXIPL and PPARGC1A to predict prognosis in FUSCC cohort, and validated prognostic ability in TCGA cohort.

After integrating all the significant clinicopathological parameters and gene expression profiles in the multivariate Cox regression models of FUSCC cohort, we generated the formula: Integrated score_(MLXIPL)_ = 2.105×pT stage (ref. T1-T2) + 1.922×pN stage (ref. N0) + 1.771×pM stage (ref. M0) + 2.413×AJCC stage (ref. I-II) + 1.934×ISUP grade (ref. 1-2) + 1.963×MLXIPL expression (ref. low) for PFS; Integrated score_(MLXIPL)_ = 1.840×pT stage (ref. T1-T2) + 1.832×pN stage (ref. N0) + 2.001×pM stage (ref. M0) + 3.434×AJCC stage (ref. I-II) + 1.764×ISUP grade (ref. 1-2) + 1.545×MLXIPL expression (ref. low) for OS. For PPARGC1A, we generated formula: Integrated score_(PPARGC1A)_ = 1.931×pT stage (ref. T1-T2) + 2.029×pN stage (ref. N0) + 1.641×pM stage (ref. M0) + 2.721×AJCC stage (ref. I-II) + 1.823×ISUP grade (ref. 1-2) + 0.524×PPARGC1A expression (ref. low) for PFS; Integrated score_(PPARGC1A)_ = 1.862×pT stage (ref. T1-T2) + 1.821×pN stage (ref. N0) + 1.912×pM stage (ref. M0) + 3.511×AJCC stage (ref. I-II) + 1.749×ISUP grade (ref. 1-2) + 0.665×PPARGC1A expression (ref. low) for OS. The AUC index of MLXIPL and PPARGC1A for the FUSCC-PFS and FUSCC-OS were 0.765, 0.768 and 0.778, 0.799, respectively (*p*<0.001; Figure 6A–6B). External validation was implemented in TCGA cohort. The AUC index of MLXIPL and PPARGC1A for the TCGA-PFS and TCGA-OS were 0.753, 0.750 and 0.748, 0.737, respectively (*p*<0.001; Figure 6C–6D). Survival curves suggested that integrated scores of *MLXIPL* and *PPARGC1A* expression significantly correlated prognosis in FUSCC cohort, and were validated significant in predicting prognosis in TCGA cohort (Supplementary Figure 4).

### Immune infiltration of MLXIPL and PPARGC1A

**Figure 7:**
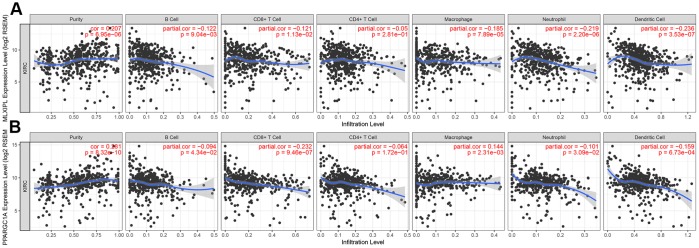
**Immune infiltration of MLXIPL and PPARGC1A.** After identifying prognostic value of MLXIPL and PPARGC1A, we performed correlation analysis between MLXIPL and PPARGC1A and immune infiltration level for ccRCC. Scatter plots were generated with partial Spearman's correlation and statistical significance. MLXIPL and PPARGC1A expression were significantly associated purity (correlation=0.207 and 0.287, respectively). In addition, elevated MLXIPL and PPARGC1A significantly correlated with B cell, CD8+ T cell, macrophage, neotrophil, and dendritic cell infiltration (p<0.05), prompting a general decline in immune infiltration level.

After identifying prognostic value of *MLXIPL* and *PPARGC1A*, we performed correlation analysis between *MLXIPL* and *PPARGC1A* and immune infiltration level for ccRCC in Figure 7. Scatter plots were generated with partial Spearman’s correlation and statistical significance. MLXIPL and PPARGC1A expression were significantly associated purity (correlation=0.207 and 0.287, respectively). In addition, elevated MLXIPL and PPARGC1A significantly correlated with B cell, CD8^+^ T cell, macrophage, neotrophil, and dendritic cell infiltration (*p*<0.05), prompting a general decline in immune infiltration level.

In Table 3, Spearman’s correlation and estimated statistical significance between MLXIPL, PPARGC1A expression and immune cell signature infiltration were displayed in detail. Correlation analysis between MLXIPL and PPARGC1A and immune cell infiltrations in ccRCC and normal samples were assessed in TCGA cohort in Supplementary Table 1. Partial correlation and correlation adjusted by tumor purity were also provided. Important signatures of a variety of immune cells include CD8^+^ T cell, T cell (general), B cell, Monocyte, tumor-associated macrophage (TAM), M1 Macrophage, M2, Macrophage, Neutrophils, Natural killer cell, Dendritic cell, Th1, Th2, Tfh, Th17, Treg, T cell exhaustion, were illustrated. (*p< 0.05; **p< 0.01; ***p< 0.001; ****p< 0.0001).

**Table 3:** Correlation analysis between MLXIPL and PPARGC1A and immune cell infiltrations for ccRCC.

**Description**	**Gene markers**	**MLXIPL**						**PPARGC1A**				
		**None**			**Purity**			**None**			**Purity**	
		**Cor**	**P**		**Cor**	**P**		**Cor**	**P**		**Cor**	**P**
CD8+ T cell	CD8A	-0.078	0.073		-0.055	0.24		-0.346	****		-0.283	****
	CD8B	-0.035	0.423		-0.009	0.853		-0.348	****		-0.283	****
T cell (general)	CD3D	-0.059	0.176		-0.036	0.444		-0.422	****		-0.358	****
	CD3E	-0.062	0.153		-0.038	0.418		-0.397	****		-0.336	****
	CD2	-0.076	0.08		-0.05	0.289		-0.393	****		-0.328	****
B cell	CD19	-0.121	**		-0.096	*		-0.325	****		-0.257	****
	CD79A	-0.155	***		-0.143	**		-0.362	****		-0.309	****
Monocyte	CD86	-0.244	****		-0.226	****		-0.266	****		-0.181	****
	CD115 (CSF1R)	-0.249	****		-0.21	****		-0.214	****		-0.139	**
TAM	CCL2	0.079	0.068		0.059	0.207		-0.134	**		-0.062	0.183
	CD68	-0.073	0.092		-0.072	0.12		-0.225	****		-0.184	****
	IL10	-0.236	****		-0.189	****		-0.24	****		-0.167	***
M1 Macrophage	INOS (NOS2)	-0.034	0.436		-0.023	0.629		0.048	0.264		0.088	0.059
	IRF5	0.221	****		0.199	****		-0.196	****		-0.173	***
	COX2 (PTGS2)	-0.284	****		-0.268	****		-0.083	0.054		-0.04	0.39
M2 Macrophage	CD163	-0.285	****		-0.241	****		-0.079	0.068		-0.033	0.474
	VSIG4	-0.294	****		-0.249	****		-0.237	****		-0.183	****
	MS4A4A	-0.285	****		-0.243	****		-0.208	****		-0.146	**
Neutrophils	CD66b (CEACAM8)	0.1	*		0.09	0.053		0.073	0.094		0.051	0.276
	CD11b (ITGAM)	-0.131	**		-0.093	*		-0.167	***		-0.094	*
	CCR7	-0.125	**		-0.109	*		-0.307	****		-0.236	****
Natural killer cell	KIR2DL1	0.147	***		0.137	**		-0.094	*		-0.1	*
	KIR2DL3	0.108	*		0.103	*		-0.085	0.051		-0.08	0.084
	KIR2DL4	0.028	0.512		0.033	0.475		-0.284	****		-0.267	****
	KIR3DL1	0.149	***		0.119	*		-0.056	0.199		-0.076	0.105
	KIR3DL2	0.133	**		0.094	*		-0.193	****		-0.211	****
	KIR3DL3	0.005	0.912		-0.016	0.738		-0.105	*		-0.081	0.082
	KIR2DS4	0.077	0.074		0.072	0.124		-0.139	**		-0.162	***
Dendritic cell	HLA-DPB1	-0.095	*		-0.062	0.184		-0.28	****		-0.213	****
	HLA-DQB1	0.056	0.195		0.085	0.069		-0.249	****		-0.18	***
	HLA-DRA	-0.143	***		-0.114	*		-0.23	****		-0.159	***
	HLA-DPA1	-0.144	***		-0.115	*		-0.247	****		-0.169	***
	BDCA-1 (CD1C)	-0.002	0.966		0.052	0.268		-0.085	*		-0.009	0.841
	BDCA-4 (NRP1)	-0.14	**		-0.121	**		0.002	0.968		0.042	0.368
	CD11c (ITGAX)	0.113	**		0.118	*		-0.207	****		-0.165	***
Th1	T-bet (TBX21)	0.106	*		0.1	*		-0.308	****		-0.291	****
	STAT4	0.027	0.532		0.023	0.623		-0.394	****		-0.356	****
	STAT1	-0.285	****		-0.269	****		-0.222	****		-0.152	**
	IFN-γ (IFNG)	-0.07	0.108		-0.051	0.272		-0.38	****		-0.325	****
	TNF-α (TNF)	-0.005	0.906		0	0.998		-0.177	****		-0.132	**
Th2	GATA3	-0.159	***		-0.128	**		-0.216	****		-0.191	****
	STAT6	0.267	****		0.234	****		-0.026	0.552		-0.057	0.218
	STAT5A	-0.124	**		-0.091	0.051		-0.251	****		-0.172	***
	IL13	0.209	****		0.162	***		-0.176	****		-0.153	***
Tfh	BCL6	-0.011	0.804		-0.052	0.262		-0.13	**		-0.156	***
	IL21	-0.131	**		-0.122	**		-0.104	*		-0.095	*
Th17	STAT3	-0.323	****		-0.29	****		-0.023	0.604		0.047	0.309
	IL17A	-0.01	0.816		-0.031	0.509		-0.026	0.545		0.02	0.667
Treg	FOXP3	-0.08	0.064		-0.064	0.168		-0.422	****		-0.346	****
	CCR8	-0.12	**		-0.095	*		-0.271	****		-0.184	****
	STAT5B	0.107	*		0.075	0.106		0.222	****		0.209	****
	TGFβ (TGFB1)	-0.237	****		-0.233	****		-0.387	****		-0.361	****
T cell exhaustion	PD-1 (PDCD1)	0.02	0.645		0.03	0.516		-0.363	****		-0.308	****
	CTLA4	-0.006	0.894		0.003	0.949		-0.309	****		-0.23	****
	LAG3	-0.03	0.489		-0.021	0.649		-0.433	****		-0.372	****
	TIM-3 (HAVCR2)	0.083	0.054		0.084	0.072		-0.01	0.822		0.055	0.234
	GZMB	0.031	0.481		0.025	0.587		-0.387	****		-0.348	****

TAM, tumor-associated macrophage; Th, T helper cell; Tfh, Follicular helper T cell; Treg, regulatory T cell; Cor, R value of Spearman’s correlation; None, correlation without adjustment. Purity, correlation adjusted by purity.

· *p*< 0.05; ** *p*< 0.01; *** *p*< 0.001; **** *p*< 0.0001.

## DISCUSSION

With the rapid development of microarray sequencing, researchers are increasingly exploring new targets and performing external validations using statistical algorithms in ccRCC [12, 16]. However, most current studies have not effectively classified and analyzed the components of cancer cells and the TME, which may markedly affect the characteristics of cancer treatment response, especially precision immunotherapy [8]. In this study, we attempted to explore TME components, extracting significant DEGs of large prognostic value to understand aggressive tumor progression in ccRCC patients. By comparing transcriptional expression profiles in 533 ccRCC patients with high versus low stromal/immune scores, a total of 77 upregulated DEGs involved in extracellular matrix components and immune response were identified. Besides significant gene penal, transcriptional *SLC27A2, G6PC, MGAM, TRPM3, PKHD1, MYL3, MAPT, SLC22A6, TRHDE, TMEM174, SLC22A8, OGDHL, SCGN, SLC51B, SLC22A12, REN, PAH, GABRG1, SLC13A2, SST, KCNJ11*, *TUBB4A* and *RGS7* expression significantly predicted overall survival for ccRCC patients. Subsequently, the expression of eight hub genes including *AGPAT9, AQP7, HMGCS2, KLF15, MLXIPL*, and *PPARGC1A* were enrolled in multivariate analysis for overall survival in ccRCC. Importantly, *MLXIPL* and *PPARGC1A* mRNA expression was significantly correlated with immune cell infiltration by Person’s correlation analysis.

Human 1-acylglycerol-3-phosphate O-acyltransferase 9 (*AGPAT9*, also known as *GPAT3* or *LPCAT1*) catalyzes the acyltransferase activity of glycerol-3-phosphate to lysophosphatidic acid [17]. Elevated *AGPAT9* expression was identified in omental adipose tissue, spleen, and lung, participating in human inflammatory stimulation and body lipid homeostasis [18]. Previous studies indicated that *AGPAT9* is involved in fatty acid metabolism, and is correlated with the TME and aggressive tumor progression [19, 20].

Aquaporin 7 (*AQP7*), a permeation protein of cell aquaporin membrane channels, promotes the transport of water and glycerol and is critical for fatty acid metabolism [21]. *AQP7* has been identified as possible major route of arsenite uptake into cells in humans [22]. Subsequently in several real-world cohorts, increased *AQP7* mRNA expression demonstrated a significant association with advanced tumor grade, stage, and lymphatic metastasis events, as well as poor prognosis in breast [23] and liver [24] cancers.

Mitochondrial 3-hydroxy-3-methylglutaryl-CoA synthase 2 (*HMGCS2*) is implicated as having oncogenetic activity in many human neoplasms [25, 26]. An integrated analysis focused on lipid metabolism and local immune response indicated *HMGGCS2, CD36*, and *GPX2* as differential hub genes of lipid metabolism in the pan-cancer immune microenvironment [26]. Transcription factor Krüppel-like factor 15 (*KLF15*) is involved in RNA polymerase II-specific DNA-binding transcription factor activity and has various functional annotations, including adipogenesis, cell cycle transition, and cell proliferation [27].

MLX-interacting protein-like (*MLXIPL*; also known as *ChREBP*) is reported to be involved in energy microenvironment homeostasis and insulin resistance [28]. In collaboration with *KLF15, MLXIPL* facilitates RNA polymerase II-specific DNA-binding transcription factor activity in glucose-activated processes. Iizuka *et al.* inferred that *MLXIPL* probably links metabolic disorders and neoplasms [29]. As a promising biological candidate reflecting the microenvironment and cancers, *MLXIPL* transitivity stimulates aerobic glycolysis by regulating glucose and lipid metabolism hallmark-related genes.

Peroxisome proliferator-activated receptor gamma coactivator-1 (*PPARGC1A*; *PGC-1α*) is a transcriptional co-regulator, and its polymorphisms are proposed as obesity metabolic regulators and to be involved in epithelial–mesenchymal transition [30, 31]. It was revealed that mitochondrial biogenesis and oxidative phosphorylation induced by PGC-1α are indispensable for migratory tumor cell metastasis [31]. Based on the PGC1α–ERRα axis, cell sensitivity to mitochondrial alterations and oxidative stress were altered, leading to perturbed invasion ability for tumor cells [31, 32].

In this current study, we focused on differential gene profiles in the TME, which in turn impact clinicopathological characteristics and aggressive tumor progression in ccRCC patients. There are several limitations of this study. First, this study failed to explore the underlying mechanisms of signaling pathways in RCC, while functional annotations and enrichment analysis were investigated in different gene panels. Second, this study set a broader threshold to avoid neglected of potential DEGs and further explored unscreened prognostic biomarkers, including *SLC27A2, G6PC, MGAM, TRPM3, PKHD1, MYL3, MAPT, SLC22A6, TRHDE, TMEM174, SLC22A8, OGDHL, SCGN, SLC51B, SLC22A12, REN, PAH, GABRG1, SLC13A2, SST, KCNJ11*, *TUBB4A* and *RGS7*, in Supplementary Figure 3, whereas many potential DEGs still failed to be investigated due to limited research scope. Third, it would be effective to validate the significance of biomarkers to predict the immune response rate in real-world clinical ccRCC cohorts receiving immunotherapy. In addition, future research needs to explore the detailed mechanism between the expression of distinct biomarkers and the progression of ccRCC and reveal the mechanism of other carcinomas.

In conclusion, after identification of stromal and immune scores using the ESTIMATE algorithm in TCGA cohort, a list of TME-related hub genes was generated. Many additional signatures that had previously been neglected were extracted. Besides significant gene penal, transcriptional *SLC27A2, G6PC, MGAM, TRPM3, PKHD1, MYL3, MAPT, SLC22A6, TRHDE, TMEM174, SLC22A8, OGDHL, SCGN, SLC51B, SLC22A12, REN, PAH, GABRG1, SLC13A2, SST, KCNJ11*, *TUBB4A* and *RGS7* expression significantly predicted overall survival for ccRCC patients. *AGPAT9, AQP7, HMGCS2, KLF15, MLXIPL*, and *PPARGC1A* exhibited significant prognostic potential, and *MLXIPL* and *PPARGC1A* were significantly correlated with immune cell signatures for ccRCC patients, thus revealing the relevance of monitoring and manipulating the TME for ccRCC prognosis and precision immunotherapies. Additionally, it would be extremely interesting to validate whether this integrated gene panel predicts both prognosis and precision immunotherapy. Further investigation might provide comprehensive insights on the potential association of the TME and ccRCC prognosis.

## MATERIALS AND METHODS

### Ethics statement

All of the study designs and test procedures were performed in accordance with the Helsinki Declaration II. Study protocols were obtained by Fudan University Shanghai Cancer Center (FUSCC) (Shanghai, China) included in this research.

### Raw biological microarray data

KIRC patients with available RNA-sequence data from TCGA database (https://tcga-data.nci.nih.gov/tcga/) were consecutively recruited in analyses [33]. The gene expression profile was measured experimentally using the Illumina HiSeq 2000 RNA Sequencing platform by the University of North Carolina TCGA genome characterization center. Level 3 data was downloaded from TCGA data coordination center, with available clinicopathological and survival data. ESTIMATE algorithm was used to calculate immune and stromal scores using "estimate" package (http://r-forge.r-project.org; repos=rforge, dependencies=TRUE) [9].

### Patients and transcriptional expression profile

Clinicopathological parameters including ISUP grade and AJCC stage in 533 ccRCC patients from TCGA were analyzed and displayed according the immune, stromal and ESTIMATE score. One-way ANOVA test were utilized to measure statistically significance. X-tile software was utilized to take the cut-off value of immune score, stromal score and ESTIMATE score, in concordance of which overall participants were divided to two groups, respectively [34]. Survival comparison between distinct three scores identified as binary variables (high vs. low) was analyzed in 533 ccRCC patients. The primary end point for patients was progression-free survival (PFS), and overall survival (OS) was the secondary end point, which was evaluated from the date of first therapy to the date of death or last follow-up. The follow-up duration was estimated using the Kaplan-Meier method with 95% confidence intervals (95% CI) and log-rank test in distinct curves. All hypothetical tests were two-sided and *P*-values less than 0.05 were considered significant in all tests.

A total of 380 ccRCC patients from the Department of Urology of Fudan University Shanghai Cancer Center (FUSCC, Shanghai, China) from April 2009 to July 2018 were consecutively recruited in analyses. Tissue samples, including ccRCC and normal tissues, were collected during surgery and available from FUSCC tissue bank.

### Identification, normalization and elucidation of DEGs

Preprocessing and normalization of raw biological data were the first step to process DNA microarray. This process removes bias from the microarray data to ensure its uniformity and integrity. Next, background correction, propensity analysis, normalization and visualization output of probe data were performed by robust multi-array average analysis algorithm in limma package [35]. Fold change > 1.5 and adj. p < 0.05 were set as the cut-offs to screen for differentially expressed genes (DEGs). Therefore, DEGs were identified based on |Log_2_FC (fold change)|<0.5849 as statistically significance.

DAVID (http://david.ncifcrf.gov, Version 6.8) online database was performed to explore the role of development-related signaling pathways in ccRCC [36]. *P*-value<0.05 was considered statistically significant. Function annotations including biological processes (BP), molecular functions (MF), and cellular component (CC) were extracted from Gene Ontology (GO) enrichment analysis [37] and Kyoto Encyclopedia of Genes and Genomes (KEGG) [38]. Hierarchical partitioning was performed using transcriptional expression profiles of selected positively-regulated DEGs in a heat map. Color gradients suggest high (red) or low (blue) expression level.

### Protein-protein interaction (PPI) network and functional annotations

In this study, Search Tool for the Retrieval of Interacting Genes (STRING; http://string-db.org) (version 10.0) online database was used to predict PPI network of significantly positive DEGs and analyze the degree of interactions between proteins [39]. Statistically significant edger was considered as interaction combined score>0.4 (medium confidence). Cytoscape (version 3.5), an open-access bioinformatics software platform providing the possibility of molecular maps, was utilized to visualize interactive network data [40]. Molecular Complex Detection (MCODE) (version1.4.2) is a plug-in for Cytoscape used for clustering a given network based on topology to find densely connected regions [41], which is able to identify the most significant module in the PPI networks with selection as follows: MCODE scores>5, degree cut-off=2, node score cut-off=0.2, Max depth=100 and k-score=2.

ClueGO is a Cytoscape plug-in that visualizes the non-redundant biological terms for large clusters of genes in a functionally grouped network [42]. GO: biological process and KEGG pathways analysis of selected hub genes were enrolled and visualized using ClueGO (version 2.5.3) and CluePedia (version 1.5.3), a functional extension of ClueGO, plug-in of Cytoscape [43].

### Real-time quantitative PCR analysis

Total RNA sequence was extracted using TRIzol^®^ reagent (Invitrogen Life Technologies, USA) from 380 paired tumor and para-carcinoma normal samples according to manufacturer’s protocol. Then, total RNA from each sample was reverse transcribed to cDNA using the PrimeScriptTM RT reagent Kit (Takara Bio Inc., Japan). Primers were diluted in ddH_2_O with SYBR Green PCR Master Mix (Applied Biosystems, Japan) according to the manufacturer’s instructions. The forward and reverse PCR primers for MLXIPL (ChREBP) are 5′- AAAACTGGGTTCTGGGTGTTC -3′ and 5′- AGGGAGTTCAGGACAGTTGG -3′, respectively. The forward and reverse primers for PPARGC1A are 5′- TGAACTGAGGGACAGTGATTTC -3′ and 5′- CCCAAGGGTAGCTCAGTTTATC -3′, respectively. Transcriptional expression was determined and normalized to β-Actin expression, and then analyzed by the -ΔΔCt method. Relative expression in ccRCC was represented using the ratio in Tumor/Normal tissues (T/N) or Normal/Tumor tissues (N/T).

### Hub genes selection and statistics analysis in two cohorts

The most co-regulated hub genes penal were strived from MCODE. Clinical and pathological parameters and transcriptional expression profiles of hub genes in 533 ccRCC patients from TCGA cohort and 380 patients from FUSCC cohort were analyzed and displayed. Expression of hub genes was respectively identified as binary variables (high vs. low) referring to median expression taking the cut-off value of each hub genes. Partial likelihood test from Cox regression analysis was developed to address the influence of independent factors on PFS and OS. The follow-up duration was estimated using the Kaplan-Meier method with 95% confidence intervals (95%CI) and log-rank test in separate curves. Student’s t tests were utilized to compare differential hub genes expression between tumor and normal tissues (*p<0.05, **p<0.01, ***p<0.001, ****p<0.0001). Univariate and multivariate analysis were performed with Cox logistic regression models to find independent variables, including stromal score (ref. Low), immune score (ref. Low), pT stage (ref. T1-T2), pN stage (ref. N0), pM stage (ref. M0), AJCC stage (ref. I-II), ISUP grade (ref. 1-2), and each hub genes expression (ref. Low).

Integrated score was identified as sum of the weight of each significant hub gene and significant clinicopathological prognostic indicators. Tumor Immune Estimation Resource (TIMER, https://cistrome.shinyapps.io/timer/) and GEPIA (http://gepia.cancer-pku.cn/detail.php) was used to perform comprehensive correlation analysis between tumor-infiltrating immune cells signatures and selected hub genes. All hypothetical tests were two-sided and *p*-values less than 0.05 were considered significant in all tests. All of these statistical analyses were performed in R or corresponding R packages survival and survminer.

### Ethics approval

The Ethics approval and consent to participate of the current study was approved and consented by the ethics committee of Fudan University Shanghai Cancer center.

## Supplementary Material

Supplementary Figures

Supplementary Table 1
