# Supplementary material for: Prognostic value and immune infiltration of novel signatures in clear cell renal cell carcinoma microenvironment
Source: Aging (Albany NY). 2019 Sep 7;11(17):6999–7020. doi: 10.18632/aging.102233 (PMC6756904; doi:10.18632/aging.102233)
Supplement: Supplementary Figures [file aging-11-102233-s002.pdf]

SUPPLEMENTARY FIGURES

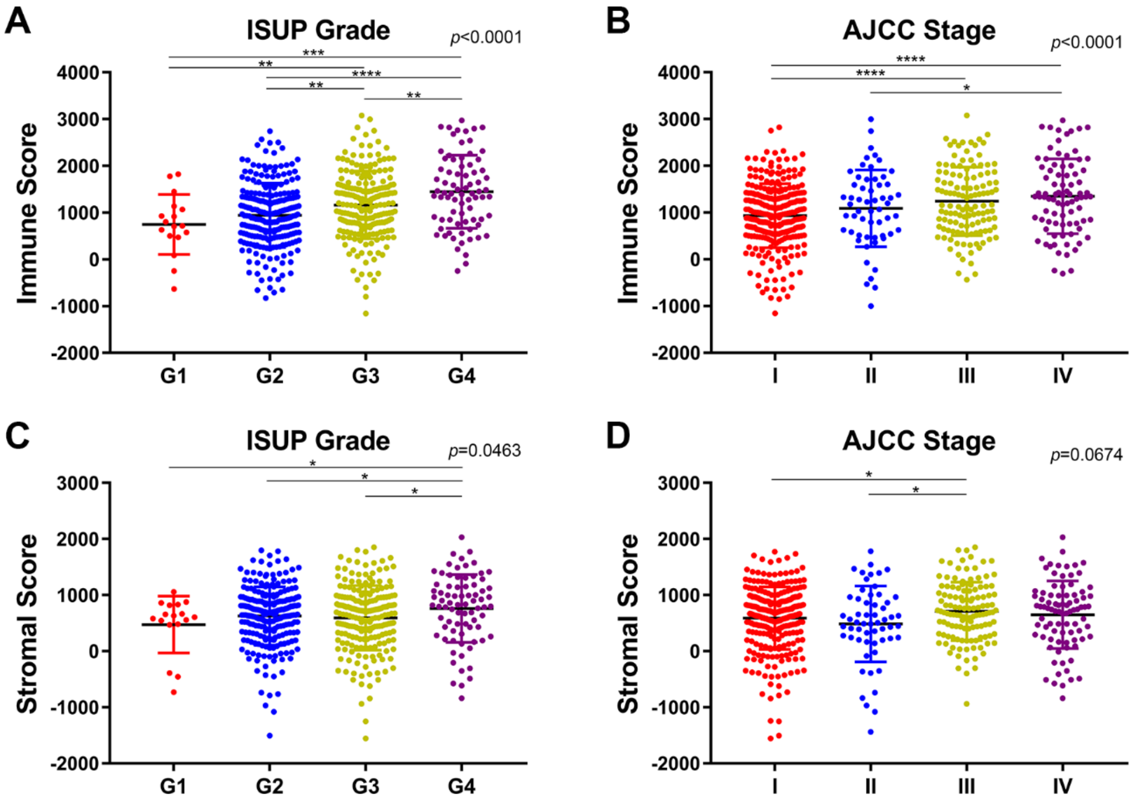

Supplementary Figure 1. (**A**–**B**) Immune score indicated significant prognostic implications, associated with elevated ISUP grade and AJCC stage (p<0.0001). (**C**–**D**) Stromal score significantly correlated with advanced ISUP grade (p=0.0463), while showed no association with AJCC stage (p=0.0674).

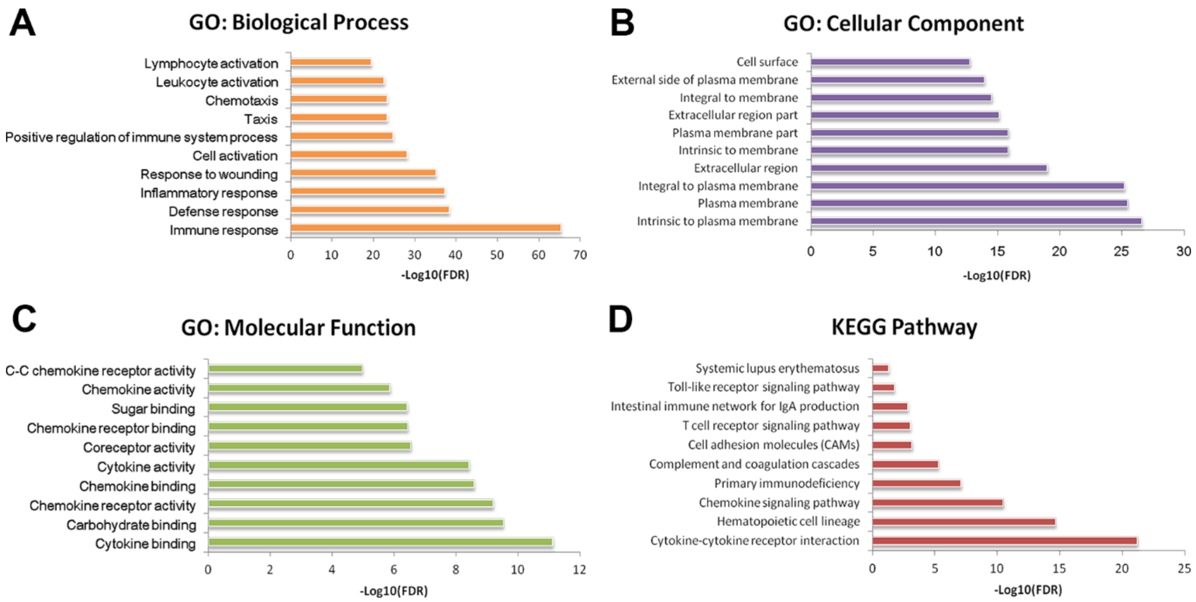

Supplementary Figure 2. After -Log (FDR) sorting, we listed the top 10 function annotations of each part. DEGs were mostly enriched in immune defense, plasma membrane, cytokine binding and cytokine-cytokine receptor interaction.

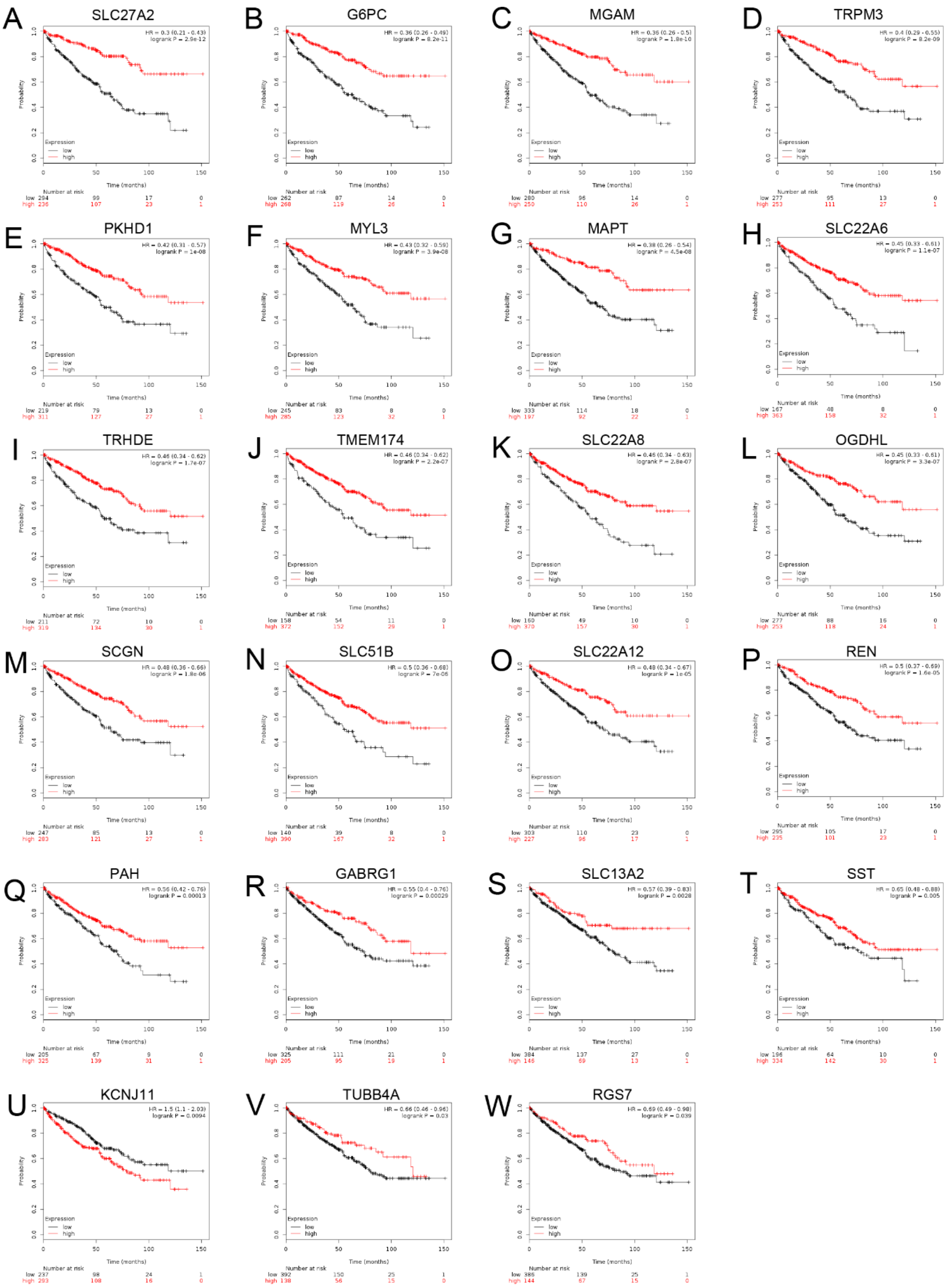

Supplementary Figure 3. Survival curves of other nodes of 77 commonly up-regulated DEGs were illustrated. It suggested that decreased SLC27A2, G6PC, MGAM, TRPM3, PKHD1, MYL3, MAPT, SLC22A6, TRHDE, TMEM174, SLC22A8, OGDHL, SCGN, SLC51B, SLC22A12, REN, PAH, GABRG1, SLC13A2, SST, KCNJ11 significantly correlated with poor OS, while elevated TUBB4A and RGS7 expression significantly predicted poor prognosis ($p<0.05$).

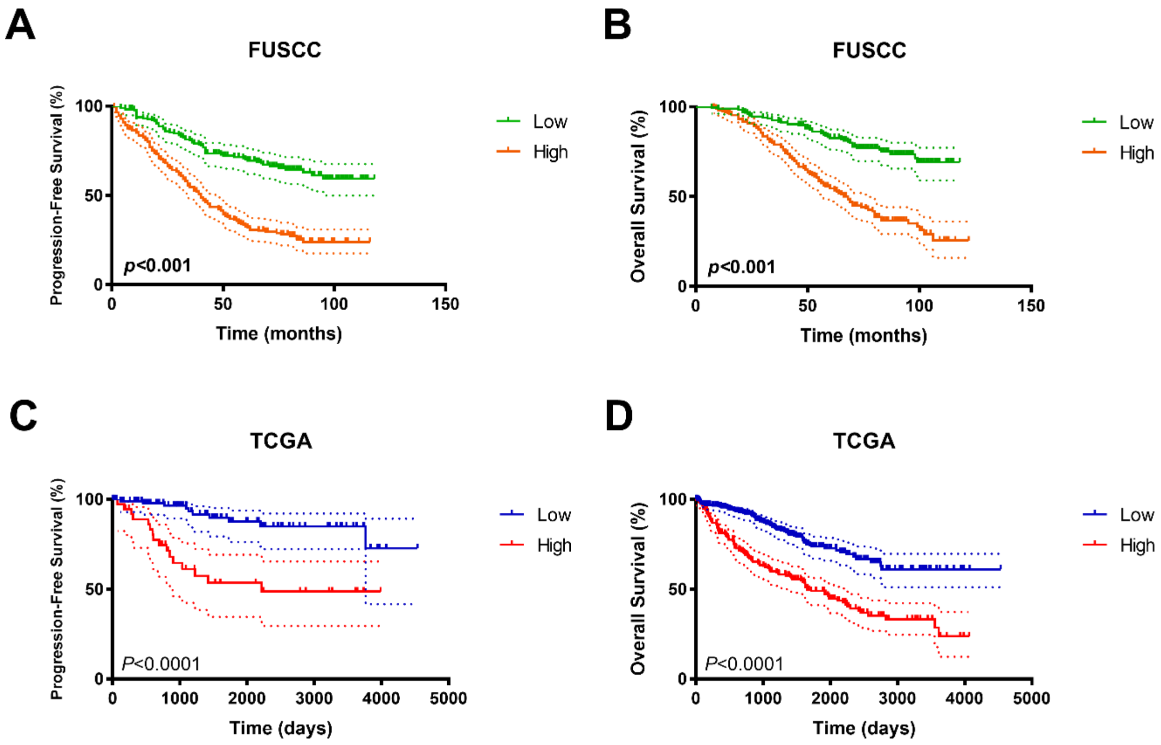

Supplementary Figure 4. Survival curves suggested that integrated scores of MLXIPL and PPARGC1A expression significantly correlated prognosis in FUSCC cohort, and were validated significant in predicting prognosis in TCGA cohort.
