# Supplementary material for: Prognostic value and immune infiltration of novel signatures in clear cell renal cell carcinoma microenvironment
Source: Aging (Albany NY). 2019 Sep 7;11(17):6999–7020. doi: 10.18632/aging.102233 (PMC6756904; doi:10.18632/aging.102233)
Supplement: Supplementary Table 1 [file aging-11-102233-s001.pdf]

SUPPLEMENTARY TABLE

Supplementary Table 1. Correlation analysis between MLXIPL and PPARGC1A and immune cell infiltrations in ccRCC and normal samples using GEPIA.

Description	Gene markers	MLXIPL				PPARGC1A			
		Tumor		Normal		Tumor		Normal	
		R	P	R	P	R	P	R	P
CD8+ T cell	CD8A	-0.12	**	0.06	0.62	-0.15	***	-0.47	****
	CD8B	-0.11	*	0.2	0.086	-0.16	***	-0.42	***
T cell (general)	CD3D	-0.14	**	0.022	0.86	-0.23	****	-0.46	****
	CD3E	-0.14	***	0.057	0.63	-0.22	****	-0.48	****
	CD2	-0.14	**	0.051	0.67	-0.2	****	-0.48	****
B cell	CD19	-0.043	0.33	-0.053	0.66	-0.047	0.28	-0.37	**
	CD79A	-0.12	**	-0.062	0.6	-0.12	**	-0.37	**
Monocyte	CD86	-0.21	****	-0.05	0.68	-0.14	**	-0.37	**
	CD115 (CSF1R)	-0.17	****	0.098	0.41	-0.12	**	-0.32	**
TAM	CCL2	0.012	0.78	-0.34	***	-0.059	0.17	-0.17	0.15
	CD68	-0.096	*	0.35	***	-0.12	**	-0.41	***
	IL10	-0.15	***	-0.17	0.16	-0.097	*	-0.019	0.11
M1 Macrophage	INOS (NOS2)	0.019	0.67	0.034	0.78	0.061	0.16	0.18	0.13
	IRF5	0.21	****	-0.6	****	-0.095	*	0.35	**
	COX2 (PTGS2)	-0.076	0.082	-0.35	***	-0.01	0.82	-0.02	0.87
M2 Macrophage	CD163	-0.22	****	-0.018	0.88	-0.12	**	-0.25	0.034
	VSIG4	-0.19	****	-0.016	0.89	-0.1	*	-0.36	**
	MS4A4A	-0.19	****	0.089	0.46	-0.12	**	-0.35	**
Neutrophils	CD66b (CEACAM8)	-0.002	0.97	-0.11	0.37	-0.011	0.8	0.3	**
	CD11b (ITGAM)	0.008	0.85	0.14	0.23	0.011	0.8	-0.34	**
	CCR7	-0.12	**	-0.047	0.69	-0.14	***	-0.33	**
Natural killer cell	KIR2DL1	0.12	**	0.14	0.25	-0.08	0.066	-0.19	0.12
	KIR2DL3	0.11	*	0.21	0.084	-0.092	*	-0.26	*
	KIR2DL4	-0.046	0.29	0.22	0.062	-0.12	**	-0.24	*
	KIR3DL1	0.056	0.2	0.16	0.19	-0.063	0.15	-0.27	*
	KIR3DL2	0.065	0.14	0.16	0.17	-0.12	**	-0.27	*
	KIR3DL3	-0.073	0.096	-0.053	0.66	0.001	0.98	0.024	0.84
	KIR2DS4	0.032	0.47	0.084	0.48	-0.077	0.078	-0.15	0.2
Dendritic cell	HLA-DPB1	-0.15	***	-0.1	0.39	-0.18	****	-0.36	**
	HLA-DQB1	-0.042	0.34	-0.27	*	-0.17	****	-0.28	*
	HLA-DRA	-0.17	****	-0.14	0.26	-0.17	****	-0.33	**
	HLA-DPA1	-0.15	***	-0.051	0.67	-0.15	****	-0.29	*
	BDCA-1 (CD1C)	-0.052	0.25	-0.066	0.58	-0.036	0.41	-0.35	**
	BDCA-4 (NRP1)	-0.072	0.1	0.13	0.26	0.025	0.57	-0.31	**
	CD11c (ITGAX)	0.079	0.072	0.12	0.32	-0.062	0.16	-0.29	*
Th1	T-bet (TBX21)	0.067	0.12	0.2	0.094	-0.19	****	-0.35	**
	STAT4	-0.038	0.38	0.068	0.57	-0.25	****	-0.4	***
	STAT1	-0.19	****	-0.52	****	-0.076	0.081	0.084	*
	IFN-γ (IFNG)	-0.11	*	0.002	0.99	-0.11	**	-0.28	*

	TNF-α (TNF)	0.006	0.9	-0.21	0.079	-0.069	0.11	-0.13	0.29
Th2	GATA3	-0.082	0.061	-0.53	****	0.044	0.32	0.53	****
	STAT6	0.21	****	-0.3	**	0.017	0.69	0.055	0.65
	STAT5A	-0.13	***	0.13	0.29	-0.065	0.14	-0.024	0.84
	IL13	0.095	*	-0.14	0.25	-0.1	*	-0.18	0.14
Tfh	BCL6	0.021	0.63	-0.57	****	-0.025	0.57	0.17	0.16
	IL21	-0.11	***	0.007	0.95	-0.067	0.13	-0.13	0.27
Th17	STAT3	-0.099	*	-0.53	****	0.049	0.27	0.32	**
	IL17A	-0.057	0.19	-0.006	0.96	-0.024	0.58	-0.15	0.22
Treg	FOXP3	-0.14	***	-0.12	0.33	-0.23	****	-0.16	0.18
	CCR8	-0.12	**	0.24	*	-0.15	***	-0.27	**
	STAT5B	0.1	*	0.024	0.84	0.17	****	0.24	*
	TGFβ (TGFB1)	-0.18	****	-0.28	*	-0.2	****	-0.056	0.64
T cell exhaustion	PD-1 (PDCD1)	-0.088	*	0.13	0.29	-0.14	**	-0.43	***
	CTLA4	-0.096	*	-0.018	0.88	-0.16	***	-0.25	0.032
	LAG3	-0.11	*	-0.36	**	-0.14	***	0.013	0.91
	TIM-3 (HAVCR2)	0.072	0.1	0.41	***	-0.047	0.29	-0.39	***
	GZMB	-0.039	0.37	0.15	0.2	-0.21	****	-0.36	**

TAM, tumor-associated macrophage; Th, T helper cell; Tfh, Follicular helper T cell; Treg, regulatory T cell; Cor, R value of Spearman's correlation; None, correlation without adjustment. Purity, correlation adjusted by purity.

* $P < 0.05$; ** $P < 0.01$; *** $P < 0.001$; **** $P < 0.0001$.
